# Influence of critical thinking disposition on the learning efficiency of problem-based learning in undergraduate medical students

**DOI:** 10.1186/s12909-018-1418-5

**Published:** 2019-01-03

**Authors:** Dan Pu, Juhua Ni, Demao Song, Weiguang Zhang, Yuedan Wang, Liling Wu, Xian Wang, Yun Wang

**Affiliations:** 1 Office of Education, Peking University School of Basic Medical Sciences, Beijing, 100083 China; 2 Department of Biochemistry and Molecular Biology, Peking University School of Basic Medical Sciences, Beijing, 100083 China; 3 Department of Physiology and Pathophysiology, Peking University School of Basic Medical Sciences, Beijing, 100083 China; 4 Department of Anatomy, Peking University School of Basic Medical Sciences, Beijing, 100083 China; 5 Department of Immunology, Peking University School of Basic Medical Sciences, Beijing, 100083 China; 6 Department of Neurobiology, Peking University School of Basic Medical Sciences, Beijing, 100083 China

**Keywords:** Personal characteristics, Problem-based learning, Critical thinking, Learning efficiency

## Abstract

**Background:**

Problem-based learning (PBL), a pedagogical approach, is widely accepted in medical education. Manipulated by many factors, the internal motivation of learner is the most crucial determinant that affects the nature of the outcome, in which the influences of critical thinking (CT) remained elusive.

**Methods:**

One hundred two third-year undergraduate medical students at Peking University were involved in this study. A Chinese version of the Critical Thinking Disposition Inventory (CTDI-CV) was used to assess the CT disposition, and the performance scores of students in PBL tutorials were compiled. A parametric bivariate correlation analysis was performed between the students’ CT scores and their PBL average scores. The PBL scores were compared between the strong and weak CT disposition groups using independent t-test. The analysis of numerical data was conducted using SPSS 16.0.

**Results:**

CT disposition of third-year undergraduate medical students at Peking University was at a positive level, with an average score of 297.72. The total CT scores had a positive correlation with the scores of the PBL performance and its five dimensions significantly. In the majority, students with Strong-CT disposition obtained higher scores in PBL tutorials compared with students with Weak-CT disposition. The performance of these two groups was significantly different in the Late-Half but not in the Early-Half PBL tutorials. Furthermore, a significant improvement was observed in the students with strong CT but not weak CT dispositions.

**Conclusion:**

CT disposition positively correlates to a students’ PBL performance. Students with stronger CT dispositions perform better in the PBL process and obtain higher scores. Our work suggested that the open-mindedness of the CT disposition is the primary factor that determines the improvement of the preparation dimensions in the PBL process.

## Background

Following the constructivist theory, the ideal learning process suggested that learner should acquire knowledge and skills actively, and the objective of education is to help students to learn how to learn. Recently, there is a broad consensus that problem-based learning (PBL), a pedagogical approach widely accepted in medical education, has a positive effect on active learning and learning outcomes [1–3]. PBL is characterized as problem-triggered, student-centered, and tutor-facilitated to achieve active lifelong learning [1, 4–6]. Comparing to graduates from traditional curriculum schools, graduates from active learning curriculum schools rated themselves higher not only on the typical PBL-related competencies, for example, the abilities of interpersonal communication, problem-solving, self-directed and lifelong learning, as well as information gathering, but also on the more general work-related skills which are deemed as the crucial aspect for success in professional practice, such as writing reports, presenting papers, doing research, producing new ideas, being able to work independently, being efficient and being able to work under pressure [7]. Schools adopting active-learning curricula had higher graduation rate than schools with conventional curricula, students graduated earlier than their counterparts from conventional schools [8]. Overall, PBL aims to foster the ability of interdisciplinary knowledge application, self-directed learning, critical thinking, communication and collaborative skills, teamwork, information searching and management, and professional values. Thus, PBL has become a modern-day educational strategy, particularly in medical education [6, 9, 10].

Many factors influence the outcomes of PBL, including the quality of the scenario [11–13], the type of learning materials [14, 15], the behaviors of the tutors or facilitators [16, 17], and the participation of the group members [18, 19]. In the point of fact, the most crucial factor of PBL is the internal motivation of the learner. It is generally accepted by the educators that PBL is an active and immersive process in which the students must take significant responsibility for their learning. Students who understand and agree with the PBL concept, who are well aware of and implement the group dynamics, who collaborate closely with team members and who are good at transforming into self-directed learners will garner increased benefits from PBL [7, 9, 11, 18].

Critical thinking (CT) is usually viewed as a philosophical concept, referred to characteristics of the individual, personality traits or habits of mind [20]. It is a pervasive and self-rectifying human phenomenon, referring to the dispositions and skills revealing what is authentic, what to believe, why it is and how it happens. A statement presented by Scriven and Paul in 1987 said, “Critical thinking is the ability to apply higher-order cognitive skills (conceptualization, analysis, evaluation) and the disposition to be deliberate about thinking (being open-minded or intellectually honest) that lead to action that is logical and appropriate” [21]. The consensus regarding critical thinking characterizes it as a self-adjusting process of judging what to believe and what to do in a given context [22]. The disposition toward CT was the consistent internal motivation to engage in problem solving and decision making.

The consensus was reached that skills and dispositions of critical thinking are positively correlated with the internal motivation to think. CT plays a central role in one’s learning and working, particularly in addition to medical education that transitions from knowledge-based curricula to competency-based curricula. It is crucial for students to learn and work further critically to evaluate existing knowledge and information. World Federation for Medical Education had considered critical thinking as one of the medical training standards. Critical thinking is vital to a health professional’s competence to assess, diagnose and care for patients correctly and effectively. Moreover, the essential requirements issued by the Institute for International Medical Education addressed personal judgments based on the ability to critically collect, analyze, evaluate and apply all sources of information and thus remain competitive in the challenging healthcare environment [23].

CT is being recognized as a liberating force in education, and a powerful resource in one’s life, increasingly exciting findings were rising in measuring and developing the students’ CT. Numerous studies suggested that CT has a definite relationship with the acquirement of knowledge and the development of professional values and clinical judgments [24–26]. Studies by Chang et al. indicated that the ability of critical thinking has a positive correlation with nursing competence significantly [24]. Ghazivakili et al. found that critical thinking correlated with academic performance substantially [25]. Several other studies also revealed the relationship between critical thinking and academic success of medical professionals, such as GPA, course grade and certification exam [26–28].

While previous studies most concentrated on following aspects: 1) how the CT disposition was in students, residents or working staffs; 2) the relationship between the CT dispositions and psychological characteristic, such as personalities, emotional intelligence, learning models, or ego resiliency; 3) whether the CT dispositions improved or not after some kinds of curriculum or education reform; 4) the relationship of CT disposition with the academic or career success. Even among the researches in respecting to the relationship of CT and PBL, most studies were concentrated on the variation of students’ CT dispositions or skills before and after PBL, or the different models and elements of PBL [26, 27, 29]. Similarly, the conclusion was reached that PBL could develop the critical thinking ability of students. However, no studies have investigated how CT and its subscales influence students’ performance in the PBL process, namely, all behaviors in PBL process, included the preparations and input, the knowledge, the skills and the attitudes.

The objective of this study is to explore the relationship of CT disposition and students’ performance in PBL and to address the following issues: 1. Does CT disposition correlate to students’ performance in PBL? 2. Is CT disposition crucial to PBL performance? 3. How does CT disposition impact the students’ performance in PBL tutorials?

## Methods

### Participants

All participants were third-year undergraduate medical students at Peking University who had finished 12 pre-clinical subjects, including anatomy, biochemistry, immunology, and microbiology. The participants also had undergone two introductory PBL tutorials and were familiar with the PBL procedures.

### PBL procedures

All students underwent eight PBL tutorials during one semester in purpose-built classrooms. Each tutorial group consisted of 12 or 13 students facilitated by one PBL tutor. The group was randomly re-assigned every two tutorials.

For each PBL tutorial, students and their tutor met three times over 2 weeks, for a total of 10 hours. Before each PBL case, students were unaware of the topic and contents. They were introduced to the scenario in the first class and were expected to discuss and ascertain what is known, what is unknown, what should be searched and what should be learned. Afterward, students would search and learn the related information, summarize the notes, submit their homework on their own and browse classmates’ homework, by themselves or with their classmates. In the second class, students would discuss and share information and more scenario would be assigned from tutors where they were requested to repeat the reflective procedures after class. After the third class, they were required to review and summarize all learning issues, and gather all materials and summarize the whole case at the end of the tutorial.

### Evaluation of students’ performance in the PBL tutorials

The performance evaluation form was first developed by our experts in assessments of medical education, based on literature review [30–32] and curriculum reform goal of PBL. According to literature, some evaluation form constructed by five aspects, that is the application of knowledge base, clinical reasoning and decision making skills, self-directed learning, collaborative work and attitude during discussion and professionalism. Some included four aspects, that is group skills, learning skills, reasoning and feedback. Some PBL tutorial assessment included four aspects, that is participation and communication skills, cooperation / team-building skills, comprehension / reasoning skills and knowledge / information gathering skills.

The objectives of PBL in our school included: application basic medical sciences for analysis of disease pathogenesis or treatment, cultivation of self-directed, active and life-long learning, promotion of team working spirit, promotion the expression and communication, development of critical thinking dispositions and skills, and development of appropriate professional attitudes and behaviors.

Therefore, combined the literature and our PBL outcomes, the student performance evaluation form consisted of five domains, which is participation, preparation, communication skills, critical discussion, and teamwork.

Students were evaluated by their tutors immediately after the case learning. Each dimension divides into five levels and accounts for 20 scores. The standard of performance is characterized in Table 1.

1. The **participation** dimension measures one’s passion for learning and the attitude towards PBL. Students are expected to attend every class, to submit their homework, and to read through all the learning materials shared by group members on time.
2. The **preparation** dimension measures one’s input on learning. Students are obligated to prepare all learning issues and to examine related information after class and submit homework online.
3. The **communication skills** dimension measures the ability to communicate with the group members. Students should express their thoughts precisely and concisely in an appropriate manner.
4. The **critical discussion** dimension measures the extent of participation and contribution in the group learning. Students should learn the references actively and critically to make comments using substantial evidence and innovative thinking.
5. The **teamwork** dimension measures the participation in group learning and the collaboration with other members. Students should accord sufficient respect to their colleagues and tutor and work together with great enthusiasm.

**Table 1:** Standard of performance and its corresponding scores

Level	1	2	3	4	5
Standard of performance	Bad	Poor	Moderate	Good	Excellent
Scores	2, 4, 6	8, 10	12	14, 16	18, 20

### Group classification

To investigate whether and how CT disposition influences the PBL performance in tutorials, students were divided into several groups. According to the extreme grouping method and the total CT scores, students with total CT scores in the top 27% of all students were defined as Strong-CT Students, and those whose total CT scores were in the bottom 27% were defined as Weak-CT Students.

Overall, eight successive PBL tutorials were conducted in one semester; the first four tutorials were classified as Early-Half PBL tutorials, and the remaining four tutorials were classified as Late-Half PBL tutorials.

### Instruments for CT disposition assessment

The critical thinking disposition was usually measured by the California Critical Thinking Disposition Inventory (CCTDI) which was first developed by Facione in 1994 [22]. In 2004, Peng et al. in The Hong Kong Polytechnic University translated and modified CCTDI in consideration of Chinese culture, developed a Chinese version of critical thinking disposition inventory (CTDI-CV) [33]. After that, most studies in China used this CTDI-CV to assess the critical thinking disposition [29, 34]. The CTDI-CV is a standardized, six Likert multiple choice test that consisted of 70 items, which could be divided into seven subscales: truth-seeking, open-mindedness, analyticity, systematicity, CT self-confidence, inquisitiveness, and cognitive maturity. Each subscale had 10 items, and its score ranged from 10 to 60. A total score above 280 and subscale scores above 40 were considered as positive CT disposition. A total score below 210 and subscale scores below 30 were considered as negative CT disposition.

Students were allowed to finish the questionnaire via an internet website, where the data were collected from their submission.

### Statistical analysis

This study was quantitatively researched, and the numerical data were analyzed by SPSS 16.0. A *P*-value < 0.05 was considered statistically significant.

## Results

### The general descriptive analysis of CTDI-CV showed students were at a positive CT level

Among a total of 168 students at Peking University, 102 (60.71%) students finished the CTDI-CV test. The CT scores ranged from 236 to 370, with a mean score of 297.72 and a standard deviation of 29.80. By assigning the index score to 280, scores above 280 were defined as a positive attitude, and the scores below 210 were defined as a negative attitude. The result showed that the CT disposition of third-year undergraduate medical students at Peking University was at a positive level on average. An analysis of seven subscale dimensions also indicated that approximately 50% of students were at a positive level (Table 2).

**Table 2:** Descriptive analysis of CTDI-CV

Subscales	Mean ± SD	Max	Min	Positive attitude (%)	Negative attitude (%)
Truth Seeking	41.19 ± 5.63	56	26	54.90	1.96
Open-mindedness	42.89 ± 4.91	52	32	67.64	0.00
Analyticity	44.21 ± 5.54	57	32	73.53	0.00
Systematicity	41.57 ± 5.65	57	30	54.90	0.00
CT self-confidence	41.13 ± 7.34	60	23	49.01	2.94
Inquisitiveness	44.19 ± 5.88	58	29	71.57	0.98
Cognitive maturity	42.55 ± 4.64	55	27	69.61	0.98
Total Scores	297.72 ± 29.80	370	236	70.59	0.00

### A significant positive association was observed between CT dispositions and PBL performance

To investigate whether CT disposition correlates with PBL performance among the students, a parametric bivariate correlation analysis was performed between the students’ CT scores and their eight successive PBL average scores. The results showed that the total CT scores had positive correlations significantly with the scores of the PBL performance and all five dimensions. Among the seven subscales, the scores of open-mindedness, analyticity, CT self-confidence and inquisitiveness showed positive correlations with the PBL total scores. In addition, open-mindedness, analyticity and CT self-confidence showed positive correlations with almost all dimensions of the PBL performance (Table 3).

**Table 3:** Pearson correlation coefficients between CT disposition and PBL performance calculated using parametric bivariate correlation analysis (*n* = 102)

CT/ CT Subscales	PBL performance					
	PBL score	Participation	Preparation	Communication skills	Critical discussion	Team working
Total Score	0.264^**^	0.266^**^	0.260^**^	0.224^*^	0.231^*^	0.211^*^
Truth Seeking	0.161	0.153	0.198^*^	0.110	0.163	0.096
Open-mindedness	0.245^*^	0.261^**^	0.226^*^	0.228^*^	0.180	0.226^*^
Analyticity	0.230^*^	0.198^*^	0.206^*^	0.197^*^	0.201^*^	0.260^**^
Systematicity	0.177	0.168	0.234^*^	0.126	0.134	0.140
CT self-confidence	0.272^**^	0.292^**^	0.242^*^	0.225^*^	0.245^*^	0.221^*^
Inquisitiveness	0.195^*^	0.203^*^	0.1543	0.163	0.170	0.184
Cognitive maturity	0.073	0.086	0.079	0.108	0.090	−0.067

^*^P < 0.05 ^**^P < 0.01

### CT disposition plays a crucial role in students’ performance in the PBL process

To investigate whether CT disposition is crucial to students’ performance in the PBL process, the PBL average scores and the scores in the Early-Half or Last-Half PBL were compared between the two extreme CT disposition groups.

### First, students with strong CT dispositions obtained higher scores in PBL tutorials

When the eight PBL tutorial average scores were compared between the Strong-CT students and Weak-CT students, significant differences in the total scores and the three dimensions of PBL performance between the two groups were found (Table 4).

**Table 4:** Significant difference in performance in PBL tutorials between Strong-CT students and Weak-CT students analyzed using independent t-test

	Scores (Mean ± SD)		
	Strong-CT students (*n* = 28)	Weak-CT students (*n* = 30)	*P* value
PBL score	89.39 ± 2.39	87.80 ± 2.27	0.011^*^
Participation	17.89 ± 0.63	17.48 ± 0.57	0.012^*^
Preparation	17.94 ± 0.48	17.66 ± 0.46	0.025^*^
Communication skills	17.80 ± 0.54	17.52 ± 0.55	0.054
Critical discussion	17.85 ± 0.56	17.49 ± 0.54	0.017^*^
Team working	17.90 ± 0.42	17.64 ± 0.40	0.017^*^

^*^P < 0.05

### Second, the performance of these two groups was not different in the early-half PBL but was significantly different in the late-half PBL

It was observed that the average scores of the PBL and the five dimensions in the two groups were not different in the Early-Half PBL tutorials. However, in the Late-Half PBL tutorials, students with strong CT dispositions had higher PBL scores than students with weak CT dispositions (Table 5).

**Table 5:** Significant performance differences were observed in Late-Half PBL tutorials between Strong-CT students and Weak-CT students but not in Early-Half PBL tutorials. The differences were analyzed using independent t-test

	Early-Half PBL tutorials (Mean ± SD)			Late-Half PBL tutorials (Mean ± SD)		
	Strong-CT group	Weak-CT group	*P* value	Strong-CT group	Weak-CT group	*P* value
PBL score	88.96 ± 2.50	88.05 ± 2.63	0.186	89.83 ± 2.65	87.54 ± 2.21	0.001^**^
Participation	17.83 ± 0.73	17.54 ± 0.62	0.108	17.96 ± 0.69	17.43 ± 0.68	0.005^**^
Preparation	17.81 ± 0.56	17.72 ± 0.65	0.551	18.08 ± 0.53	17.61 ± 0.45	0.001^**^
Communication skills	17.72 ± 0.61	17.60 ± 0.68	0.471	17.88 ± 0.66	17.44 ± 0.54	0.007^**^
Critical discussion	17.78 ± 0.62	17.58 ± 0.61	0.217	17.92 ± 0.63	17.41 ± 0.60	0.003^**^
Team working	17.81 ± 0.44	17.62 ± 0.49	0.114	17.99 ± 0.55	17.66 ± 0.48	0.018^*^

^*^P < 0.05; ^**^P < 0.01

### Third, a significant improvement was observed in students with strong CT dispositions but not in students with weak CT dispositions

The improvement scores, that is, the average scores of the Late-Half PBL after subtracting the First-Half PBL, were compared between the two extreme CT disposition groups. The average improvement score of the CT-Strong group significantly increased; conversely, the score decreased in students with weak CT dispositions, particularly in the dimensions of preparation, communication skills and critical discussion dimensions (Table 6).

**Table 6:** Significant performance improvement was observed in students with strong CT disposition but not in students with weak CT disposition. The differences were analyzed using independent t-test

	Improvement Scores (Mean ± SD)		
	Strong-CT students (*n* = 28)	Weak-CT students (*n* = 30)	*P* value
PBL scores	0.875 ± 1.96	−0.508 ± 1.74	0.006^**^
Participation	0.125 ± 0.63	−0.117 ± 0.61	0.144
Preparation	0.27 ± 0.52	− 0.11 ± 0.632	0.017^*^
Communication skills	0.161 ± 0.67	−0.158 ± 0.52	0.047^*^
Critical discussion	0.14 ± 0.55	− 0.17 ± 0.55	0.037^*^
Team working	0.18 ± 0.55	0.04 ± 0.56	0.354

^*^P < 0.05; ^**^P < 0.01

### An open-mindedness subscale of CT disposition may be the central component in students’ performance

To investigate which subscale of CT disposition is crucial to improving the students’ performance in the PBL process and to execute on the appropriate dimensions of the PBL performance, a stepwise multiple linear regression was conducted by the improvement scores. The five dimensions of the PBL performance were successively used as the dependent variable, and the subscale of the CT disposition was used as the independent variables. It was found that the open-mindedness subscale of CT disposition was the primary factor that influences the improvement of the Preparation dimensions in the PBL process (Table 7).

**Table 7:** Model summary between the PBL performance dimensions and the CT disposition subscales, analyzed using a stepwise multiple linear regression (*n* = 102)

Model	R	R square	Adjusted R square	Std. error of the estimate	Change statistics				
					R square change	F change	df1	df2	Sig F change
Preparation	0.250^a^	0.063	0.053	0.600	0.063	6.667	1	100	0.011^*^

^a^Predictors: (Constant), Open-mindedness

^*^P < 0.05

## Discussion

Our results revealed a favorable critical thinking disposition among third-year undergraduate medical students at Peking University because the average mean score on the CTDI-CV was 297.72, which was specified by Facione [22]. Following several other results from Huang L [35], students from top medical universities showed a positive attitude towards critical thinking, particularly on the subscales of analyticity and inquisitiveness. However, students showed weaker performance on the subscales of truth-seeking and CT self-confidence, which presumably ascribed to the traditional large-classroom teaching methods and the bashfulness of the Chinese culture.

A significant positive association was observed between the total CT scores and the PBL scores resulting from the correlation analysis, which meant that the CT dispositions of the learners would influence their performance during PBL tutorials.

Among the seven CT subscales, the scores of open-mindedness, analyticity, CT self-confidence, and inquisitiveness showed positive correlations with the PBL total scores, implying that students who score higher in these dispositions would be more active in the PBL process and would thus obtain higher PBL scores. Furthermore, the subscale of open-mindedness showed a positive correlation with three performance dimensions: the participation, the preparation, and the communication skills dimension; CT self-confidence showed a positive correlation with the participation and the Critical discussion dimension. According to Facione, the open-mindedness subscale addresses “*being tolerant of divergent views with sensitivity to the possibility of one’s own bias.*” Thus, students score higher in the open-mindedness disposition would prefer to collect various information, to tune in different perspectives, and to express themselves actively; this concept is demonstrated in the higher scores of the participation, the preparation, and the communication skills dimension. A stronger disposition of CT-confidence allows students to relax and participate in the learning process and discussion, which could be reflected in the higher scores of the participation and the critical discussion dimension.

More data supports our hypothesis that CT disposition plays a crucial role in students’ performance in the PBL process. First, students with strong CT dispositions obtained higher PBL scores than those with weak CT dispositions meaning that stronger CT dispositions bring students to learn more actively and to perform much better. Second, it was observed that in the Late-Half PBL tutorials, the average scores of the PBL and the five dimensions in the Strong-CT group were significantly higher than those in the Weak-CT group. On the contrary, in the Early-Half PBL tutorials, they showed no difference, which indicates that Strong-CT disposition students commit more passion in learning, and the knowledge and skills would improve as long as the process of tutorials. Finally, a significant improvement was observed in students with a strong CT disposition, which suggest that strong CT dispositions stimulate students to advance steadily. However, in the Weak-CT group, the students would behave consistently throughout whole semester without significant improvement.

Overall, our results showed that CT disposition positively correlates to a students’ PBL performance. Students with stronger CT dispositions would perform better in the PBL process and obtain higher scores. The open-mindedness of the CT disposition is the primary factor that determines the improvement of the preparation dimensions in the PBL process. Thus, the cultivation of open-mindedness plays a beneficial role in learning.

However, some limitations of this study include the following aspects. First, the 102 students involved in this study is considered as a limited sample pool. Nonetheless, these 102 students were enrolled with similar admission scores and underwent the same curricular system, which had similar features to reveal the PBL scores and CT dispositions. Second, the positive correlation between PBL scores and CT disposition could be explained by the generosity grading scales conducted by the tutors, leading to all students received similar PBL scores with low or no significance.

## Conclusions

Collectively, this study described the details of how CT disposition influence the performance in PBL tutorials. According to the consensus statement regarding critical thinking and the ideal critical thinker, *“CT is a liberating force in education and a powerful resource in one’s personal and civic life.”* Previous studies were most concentrated on how the CT disposition or its’ influence factor was, or whether the CT disposition was improved after educational reform. Although several studies mentioned that PBL might help students to improve their critical thinking, no studies considered whether and how CT and its subscales influence students’ performance in the PBL process. Our research demonstrated that CT disposition of students might also influence the learning efficiency and learning outcomes in PBL tutorials. Therefore, as a tutor in PBL tutorial or the manager in the medical curriculum, it is crucial to recognize the potential influence of critical thinking dispositions on the outcome of PBL. Systematic guiding or training of students, which would help them to acclimatize to PBL pedagogy and learning methods, should be enhanced throughout the whole course. The tutor training should be improved to promote the teaching and learning effects.

## Abbreviations

CT: Critical thinking
CTDI-CV: The Chinese version of the Critical Thinking Disposition Inventory
PBL: Problem-based learning
